# Effects of Subchronic Exposure of Diclofenac on Growth, Histopathological Changes, and Oxidative Stress in Zebrafish (*Danio rerio*)

**DOI:** 10.1155/2014/645737

**Published:** 2014-02-05

**Authors:** Eva Praskova, Lucie Plhalova, Lucie Chromcova, Stanislava Stepanova, Iveta Bedanova, Jana Blahova, Martin Hostovsky, Misa Skoric, Petr Maršálek, Eva Voslarova, Zdenka Svobodova

**Affiliations:** Department of Veterinary Public Health and Toxicology, Faculty of Veterinary Hygiene and Ecology, University of Veterinary and Pharmaceutical Sciences Brno, Palackeho 1-3, 612 42 Brno, Czech Republic

## Abstract

The aim of this study was to investigate effects of subchronic exposure to sublethal levels of diclofenac on growth, oxidative stress, and histopathological changes in *Danio rerio*. The juvenile growth tests were performed on *Danio rerio* according to OECD method number 215. Fish at the age of 20 days were exposed to the diclofenac environmental concentration commonly detected in the Czech rivers (0.02 mg L^−1^) and the range of sublethal concentrations of diclofenac (5, 15, 30, and 60 mg L^−1^) for 28 days. A significant decrease (*P* < 0.01) in the fish growth caused by diclofenac was observed in the concentrations of 30 and 60 mg L^−1^. The identified value of LOEC (lowest observed effect concentration) was 15 mg L^−1^ of diclofenac and NOEC (no observed effect concentration) value was 5 mg L^−1^ of diclofenac. We did not find histopathological changes and changes of selected parameters of oxidative stress (glutathione S-transferase, glutathione reductase) in tested fish. The environmental concentration of diclofenac in Czech rivers did not have any effect on growth, selected oxidative stress parameters (glutathione S-transferase, glutathione reductase), or histopathological changes in *Danio rerio* but it could have an influence on lipid peroxidation.

## 1. Introduction

Diclofenac represents an important drug in ambulatory care and is used to reduce pain, inflammation, and stiffness caused by many conditions, such as osteoarthritis, rheumatoid arthritis, abdominal cramps associated with menstruation, and ankylosing spondylitis. It is used worldwide and has a production volume estimated to be in the hundreds of tons annually. It is used in the form of tablets, capsules, suppositories, and intravenous solutions and in ointments and gels for dermal application [1].

Pharmaceuticals in the environment are of growing concern for their potential consequences on human and ecosystem health. The main route of entry of pharmaceuticals into the environment has been identified as effluent from sewage treatment plants and the disposal of unused drugs down the drain or with household garbage. A large number of pharmaceuticals are partially eliminated during treatment in sewage treatment plants. Low levels of pharmaceuticals (ordinarily in *μ*g L^−1^) have been detected in many countries in soil, sewage treatment plant effluents, surface waters, seawaters, groundwater, and some drinking waters [2–4]. In long-term monitoring investigations of sewage and surface water samples, diclofenac was identified as one of the most important pharmaceutically active compounds present in the water cycle [5]. It was found in groundwater samples [6, 7] and sporadically even in raw or treated drinking water [8].

Nontarget species considered to be most endangered by its action are probably aquatic organisms. Therefore, pharmaceutical effects on aquatic organisms have been investigated in acute toxicity assays [9–11]. The chronic toxicity and potential subtle effects are only marginally known, however.

The aim of this study was to investigate the effects of subchronic exposure to environmental and sublethal levels of diclofenac on growth, glutathione S-transferase, glutathione reductase, and malondialdehyde as oxidative stress parameters and on histopathological changes in *D. rerio*.

## 2. Materials and Methods

### 2.1. Experimental Design

Tests of diclofenac toxicity were performed on zebrafish (*Danio rerio*), which is one of the model organisms most commonly used in toxicity tests. The experimental fish were obtained from a commercial dealer; fish were 20 days old, average beginning weight of fish was 10 ± 2 mg, and average length was 12 ± 2 mm.

Method of subchronic toxicity tests was in compliance with OECD guidelines no. 215 (Fish, Juvenile Growth Test) [12]. The experiment was performed in four repetitions. The tests were carried out with 25 fish used for each concentration and for the control groups. Fish were placed in test aquariums and exposed to a range of sublethal concentrations of diclofenac (0.02 mg L^−1^ (environmental concentration detected in Czech rivers), 5, 15, 30, and 60 mg L^−1^). Due to the low solubility of diclofenac in water, the dissolution of the substance had to be done using ultrasound device.

The duration of these semistatic tests (the solutions were renewed at 12-hour intervals) was 28 days. Fish were fed with dried *Artemia salina* without nutshells in amount 4% of their body weight per day; the food ration was based on initial fish weight and was recalculated after 14 days. At the end of the tests fish were weighted again and their length was determined. Food was withheld from the fish 24 hours prior to weighing. During the tests, the living conditions were checked at 24-hour intervals and the mortality was recorded in each concentration. Water temperature in tests was 23 ± 2°C, oxygen saturation of water was above 60%, and pH of the water ranged from 7.6 to 8.2.

Tank-average specific growth rates were calculated using the following formula according to the OECD no. 215:

(1) $$\begin{array}{c} r_{2}=\frac{\bar{\text{lo}{\text{g}}_{e}W_{2}}-\bar{\text{lo}{\text{g}}_{e}W_{1}}}{t_{2}-t_{1}}\cdot 100, \end{array}$$

 
*r*_2_ : tank-average specific growth rate,
 
*W*_1_, *W*_2_: weight (mg) of a particular fish at times *t*_1_ and *t*_2_, respectively,
 
$\bar{\text{lo}{\text{g}}_{e}W_{1}}$: average of logarithms of the values *W*_1_ for the fish in the tank at the start of the study period,
 
$\bar{\text{lo}{\text{g}}_{e}W_{2}}$: average of logarithms of the values *W*_2_ for the fish in the tank at the end of the study period,
 
*t*_1_, *t*_2_: time (days) at start and end of study period.

### 2.2. Water Quality Parameters

The basic physical and chemical parameters of dilution water used in tests were ANC_4.5_ 4.2 mmol L^−1^; COD_Mn_ 2.8 mg L^−1^; total ammonia below the limit of determination (<0.04 mg L^−1^); NO_3_^−^ 23.48 mg L^−1^; NO_2_^−^ below the limit of determination (<0.02 mg L^−1^); Cl^−^ 18.11 mg L^−1^; and Σ  Ca^2+^ + Mg^2+^ 3.06 mmol L^−1^.

### 2.3. Statistical Analysis

Results were analysed using the statistical programme Unistat 5.1. The data were subjected to one-way ANOVA and subsequently to Dunnett's test in order to assess the statistical significance of differences in tank-average fish specific growth (*r*_2_) between test group with different concentrations and that of the control group. The estimation of the LOEC and NOEC values was based on ANOVA followed by Dunnett's test for the identification of the lowest concentration for which this difference is (or is not) significant at a 0.05 probability level. Results of oxidative stress markers were analysed by Dunnett's test too.

### 2.4. Determination of Diclofenac

During the testing (always before and after the bath change), samples for the determination of diclofenac concentration were regularly withdrawn from test tanks. Diclofenac determination in water samples was performed by high performance liquid chromatography (HPLC) with photometric detection. Water samples were filtered through a 0.45 *μ*m nylon filter (Millipore, Billerica, MA) and used for analysis. The sample volume injected into the HPLC system was 20 *μ*L. Diclofenac was separated by an isocratic elution method with acetonitrile/water 50/50 (v/v) on a Polaris C18-A column (3 *μ*m, 150 × 4.6 mm, Varian, Inc., Palo Alto, CA). The mobile phase flow rate was 1 mL min^−1^, the column temperature was 25°C, and UV detection was performed at 310 nm. Chromatographic analysis was accomplished by means of an Alliance 2695 chromatographic system (Waters, Milford, MA) with a PDA 2996 photodiode array detector (Waters, Milford, MA). Diclofenac was purchased from Sigma-Aldrich (St. Louis, MO). All solvents were of HPLC-grade purity (Chromservis, s.r.o., CZ). The detection limit for diclofenac was 11 ng mL^−1^. The limit of quantification for diclofenac was 37 ng mL^−1^. The coefficient of variation was 4.5%.

### 2.5. Histopathological Examination

Fish were fixed in 10% neutral formalin solution and processed using conventional paraffin techniques. Tissue sections were stained with haematoxylin and eosin. Histological changes in samples of skin, liver, gills, and kidney were examinated by light microscopy.

### 2.6. Oxidative Stress Examination

Whole body samples were homogenised and extracted with phosphate buffer (pH 7.2). Homogenates were centrifuged and supernatants fractions were stored in the freezer at −80°C for later analyses. The catalytic concentration of glutathione S-transferase was measured spectrophotometrically using method of Habig et al. [13]. The catalytic concentration was expressed as the nmol of the formed product per minute per mg of protein. The catalytic concentration of glutathione reductase was measured spectrophotometrically using method of Carlberg and Mannervik [14]. The catalytic concentration was expressed as the nmol of reduced nicotinamide adenine dinucleotide phosphate (NADPH) consumed per minute per mg of protein. Protein concentration was determined by a Bicinchoninic Acid Protein Essay Kit (Sigma-Aldrich) using bovine serum albumin as a standard.

To check lipid peroxidation in the samples, malondialdehyde was measured by the thiobarbituric-acid-reactive-substances (TBARS) assay described by Lushchak et al. [15]. Frozen body samples were homogenized in phosphate buffer. A 200 *μ*L of homogenate was mixed and centrifuged for 20 min at 5000 ×g. The volume of 200 *μ*L of supernatant was mixed with thiobarbituric-acid (TBA) reagent and heated for 45 min at 90°C. The absorbance of the sample was measured at 535 nm using a Varioskan Flash Spectral Scanning Multimode Reader (Thermo Fisher Scientific Inc.). The TBARS concentrations are expressed as nmol per gram wet weight of tissue [15–17].

## 3. Results

### 3.1. Subchronic Toxicity Tests

For the growth test we selected a range of 5 initial concentrations of diclofenac lower than 96h LC50. We selected concentrations 3-fold (60 mg L^−1^), 6-fold (30 mg L^−1^), 12-fold (15 mg L^−1^), and 36-fold (5 mg L^−1^) less than 96h LC50 diclofenac value and the environmental concentration of diclofenac in the Czech rivers (0.02 mg L^−1^).

### 3.2. Fish Behaviour and Mortality

In test groups of fish exposed to 0.02 mg L^−1^, 5 mg L^−1^, and 15 mg L^−1^ of diclofenac, the mortality did not exceed 5% during the 28-day experimental period. In all control tanks the mortality was 0% during the experiments. In the test groups exposed to 60 mg L^−1^ and 30 mg L^−1^, the mortality was 7.2% (for concentration 30 mg L^−1^) and 10% (for the highest concentration 60 mg L^−1^). No behaviour changes were observed.

### 3.3. Growth Rate

**Figure 1:**
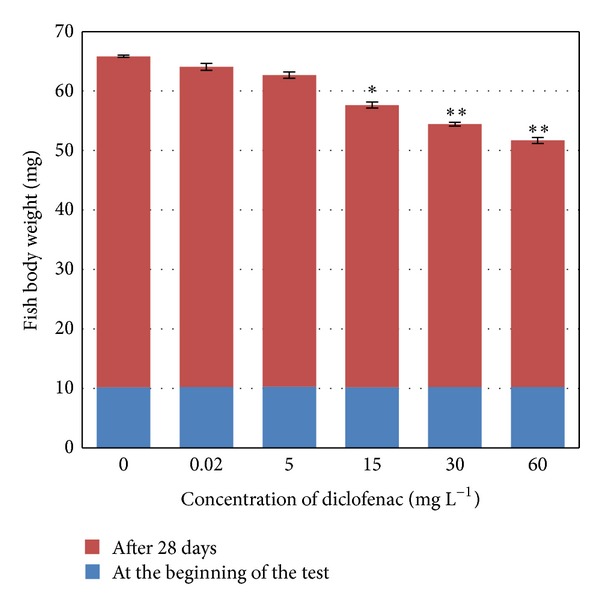
Comparison of body weight of control (0 mg L^−1^ diclofenac) and test fish (concentrations of diclofenac from 0.02 to 60 mg L^−1^) (*P < 0.05, **P < 0.01).

The overview of the results of body weight measurements before and after the series of tests (means ± standard deviations) is shown in Figure 1. The initial body weight was not significantly different among groups, but at the end of the experiment, fish weight was significantly lower in tanks with concentrations of diclofenac 15 mg L^−1^ (*P* < 0.05), 30 mg L^−1^ (*P* < 0.01), and 60 mg L^−1^ (*P* < 0.01) compared to the control group.

**Figure 2:**
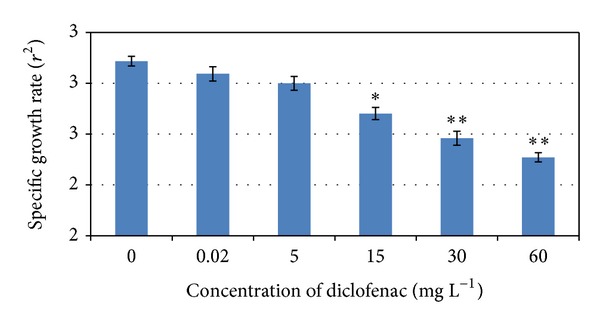
Comparison of specific growth rate and tested diclofenac concentrations (concentrations from 0.02 to 60 mg L^−1^) (*P < 0.05, **P < 0.01).

The results of specific growth rate *r*_2_ (means ± standard deviations) of the test groups in comparison with the control group are demonstrated in Figure 2. Significant decreases in fish growth caused by diclofenac concentrations of 15 mg L^−1^ (*P* < 0.05), 30 mg L^−1^ (*P* < 0.01), and 60 mg L^−1^ (*P* < 0.01) were found.

### 3.4. Body Length

**Figure 3:**
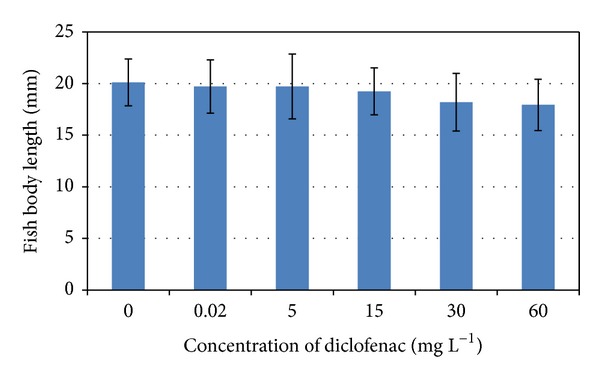
Comparison of the individual body lengths of fish (concentrations of diclofenac from 0 to 60 mg L^−1^).

The results of individual fish body length (means ± standard deviations) at the end of the experiment in comparison with control groups are presented in Figure 3. No significant decrease in individual fish body length caused by diclofenac concentration was detected.

### 3.5. Histopathological Changes

No histopathological changes in samples of skin, liver, gills, and kidney were observed.

### 3.6. Oxidative Stress

**Figure 4:**
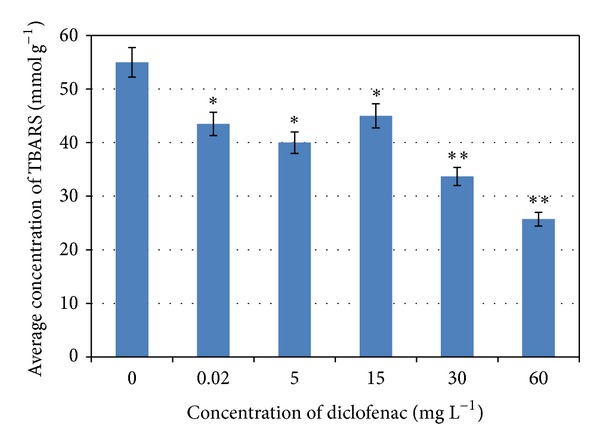
Comparison of concentrations of TBARS in fish kept in tested diclofenac concentrations (concentrations from 0.02 to 60 mg L^−1^) (*P < 0.05, **P < 0.01).

Data showed significant decrease of TBARS concentrations in fish in all tested diclofenac concentrations (Figure 4). No changes of glutathione S-transferase and glutathione reductase were observed.

The results of growth and oxidative stress parameters determined value of LOEC as 0.02 mg L^−1^ of diclofenac.

### 3.7. Validity of the Tests

Our tests met all conditions required by OECD: the mortality in the control groups below 10% (no fish died in the control tanks), the final weight of control fish in subchronic toxicity tests was higher than 150% of the initial weight, the dissolved oxygen concentrations were at least 60%, the water temperature did not differ more than ±1°C among test aquariums, and test substance concentrations were above 80% of measured initial concentration.

## 4. Discussion

Not many authors mentioned the acute effect of diclofenac on fish. Diclofenac toxicity has been monitored in zebrafish embryos, however. Hallare et al. [9] who studied diclofenac toxicity to zebrafish embryos exposed to concentrations of 1–2000 *μ*g L^−1^ found delayed hatching and hydroedema. Van den Brandhof and Montforts [10] found growth retardation, delayed hatching, and yolk sac and tail deformation in concentrations of diclofenac above 1.5 mg L^−1^. In their study 72 h EC50 was found to be 5.3 mg L^−1^. Praskova et al. [11] found 144 h LC50 = 6.11 mg L^−1^ for embryos of zebrafish and 166.6 mg L^−1^ for juvenile zebrafish.

Only a few studies were performed as chronic tests. Three-month exposure of fish (*Oryzias latipes*) to 0.001–10 mg L^−1^ of diclofenac resulted in significant decreasing trend in hatching success and delay in hatch [18]. In our study, values of LOEC (15 mg L^−1^ of diclofenac) and NOEC (5 mg L^−1^ of diclofenac) were determined.

Tissues of the rainbow trout (*Oncorhynchus mykiss*) exposed to diclofenac concentrations ranging from 1 *μ*g L^−1^ to 500 *μ*g L^−1^ over a 28-day period were investigated by histopathological methods. The highest concentrations of diclofenac were detected in the liver, followed by the kidneys and the gills [19]. Schwaiger et al. [19] found the most prominent reactions induced by diclofenac in the kidney: a severe accumulation of protein in the tubular cells, macrophage infiltration, and structural alterations (dilation, vesiculation) of the endoplasmic reticulum in the renal tubules. Furthermore, necrosis of endothelial cells in the renal corpuscles had occurred. In the liver, the most striking reactions were the collapse of the cellular compartmentation and glycogen depletion in hepatocytes. Observations made in the gills included pillar cell necrosis and hypertrophy of chloride cells; epithelium lifting had also become evident in the secondary lamellae [19]. We did not find any histopathological changes in zebrafish exposed to diclofenac concentrations up to 60 mg L^−1^ for 28 days, though.

The possibility of nephrotoxic effects of diclofenac after chronic exposure was described by Revai and Harmos [20]. In concentrations ranging from 7 to 15 *μ*g L^−1^, diclofenac exposure induced tubular necrosis in the kidneys of the rainbow trout, and hyperplasia and fusion of the villi in the intestine were detected in concentrations above 1 *μ*g L^−1^. This study demonstrates that subchronic exposure to environmental concentrations of diclofenac can lead to its interference in the biochemical functions of fish and to tissue damage, further highlighting concern about this pharmaceutical in the aquatic environment [21, 22].

Furthermore, possible effects of higher concentrations of diclofenac on fish weight demonstrated in our study should be considered. Further studies should be performed, for example, on carps to assess the possible occurrence of oxidative stress in tissues that are well-known targets of diclofenac, such as kidney.

Analysis of parameters of oxidative stress showed reduction in lipid peroxidation in fish caused by diclofenac occurrence in the water environment. To the best knowledge of the authors of this paper there are not many studies of oxidative stress on fish caused by pharmaceuticals. Our results are in agreement with Feito et al. [23] who found out a reduction of lipid peroxidation after a very short exposure (90 min) of zebrafish embryos to 0.03 *μ*g L^−1^ of diclofenac. Our observations are in agreement with Petersen et al. [24] that low concentrations of NSAIDs (nonsteroidal anti-inflammatory drugs) may protect against oxidative stress.

## 5. Conclusions

The environmental concentrations of diclofenac in Czech rivers (0.02 mg L^−1^) and surface waters of other aforementioned countries (ordinarily in *μ*g L^−1^) had no effect on growth, on histopathological changes, and on glutathione S-transferase and glutathione reductase in *D. rerio*. It was proved that diclofenac occurrence in water environment caused a decrease of lipid peroxidation in *D. rerio* fish, though.

The concentration of 15 mg L^−1^ of diclofenac (LOEC) caused the decrease in the fish growth. It may be concluded that the environmental concentration of diclofenac is lower than determined diclofenac NOEC and LOEC values.
